# Recent Advances in Repetitive Transcranial Magnetic Stimulation for the Treatment of Post‐Stroke Cognitive Impairment

**DOI:** 10.1002/cns.70702

**Published:** 2025-12-10

**Authors:** Yu Liu, Yansong Li, Ding Ding, Aiguo Xie, Jiaran Yan, Xiaojin Xu, Zihan Zhao, Jing Wu, Jing Wang, Mingfeng Qian

**Affiliations:** ^1^ Department of Neurology The Air Force Hospital of Northern Theater PLA Shenyang China; ^2^ The Air Force Hospital of Northern Theater PLA Shenyang China

**Keywords:** activities of daily living, attention function, dorsolateral prefrontal cortex, executive function, memory function, post‐stroke cognitive impairment, repetitive transcranial magnetic stimulation

## Abstract

**Background:**

Post‐stroke cognitive impairment (PSCI) is a prevalent and disabling condition with limited effective treatment options. Repetitive transcranial magnetic stimulation (rTMS) has emerged as a potential non‐invasive neuromodulation therapy. This review synthesizes recent advances in rTMS for PSCI, focusing on its mechanisms, therapeutic effects across cognitive domains, and safety profile.

**Methods:**

We summarize evidence indicating that rTMS exerts its effects by modulating cortical excitability, promoting neuroplasticity via BDNF signaling, and regulating dysfunctional brain networks, particularly the central executive and default mode networks.

**Results:**

Clinical studies demonstrate that high‐frequency stimulation, primarily targeting the dorsolateral prefrontal cortex (DLPFC), can significantly improve memory, executive function, attention, and activities of daily living (ADLs) in patients with PSCI. A favorable safety profile is reported, with mild and transient adverse effects being most common. However, significant heterogeneity in stimulation parameters (e.g., frequency, intensity, pulses) exists across studies. Current evidence suggests that ensuring a sufficient number of stimulation pulses and duration may be necessary.

**Conclusion:**

rTMS represents a promising therapeutic tool for PSCI, demonstrating benefits in key cognitive and functional domains. Future research must prioritize large‐scale, standardized randomized controlled trials to optimize stimulation protocols, confirm long‐term efficacy, and explore synergistic combinations with other rehabilitation strategies.

## Graphical abstract

This review synthesizes evidence that rTMS safely improves memory, executive function, and other cognitive domains in PSCI by modulating brain networks and neuroplasticity. Despite parameter heterogeneity, sufficient pulse dosage and treatment duration appear critical for efficacy. Future randomized controlled trials are needed to optimize protocols and confirm long‐term benefits.

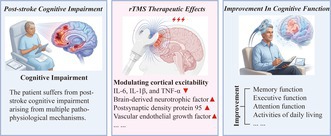

## 1 Introduction

Stroke represents the second leading cause of global mortality and a major contributor to disability worldwide. In 2019, stroke accounted for approximately 6.55 million deaths and 143 million disability‐adjusted life‐years globally [1]. While advancements in acute stroke interventions have driven measurable improvements in clinical outcomes, post‐stroke cognitive impairment (PSCI) remains a highly prevalent and clinically disabling complication [2, 3]. PSCI can significantly compromise activities of daily living, hinder vocational rehabilitation, and is strongly associated with the development of post‐stroke depression. PSCI prevalence peaks in the initial months following stroke, with acute PSCI affecting approximately 75% of patients and chronic PSCI persisting in approximately 50% of cases [4]. Spatial neglect and aphasia emerge as the most prevalent and disabling deficits [5]. PSCI significantly impairs patients' quality of life and imposes substantial burdens on families and healthcare systems. Currently, there remains a lack of high‐level evidence supporting pharmacological and psychological interventions for PSCI, underscoring an urgent need for novel therapeutic approaches.

Recent advances in noninvasive brain stimulation techniques have introduced novel therapeutic strategies for neurological disorders. Among these, repetitive transcranial magnetic stimulation (rTMS) has emerged as a prominent neuromodulatory approach for conditions such as Alzheimer's disease (AD), Parkinson's disease (PD), multiple sclerosis (MS), depression, vascular dementia, and stroke [6, 7, 8, 9]. Over recent decades, rTMS has been increasingly utilized in the management of PSCI, with clinical studies demonstrating significant enhancements in various cognitive domains, such as global cognition, memory, attention, and executive function [10, 11, 12]. These findings suggest that rTMS may serve as an effective non‐invasive neuromodulation tool for treating PSCI.

However, a significant challenge remains: there is no unified standard for target selection and stimulation parameters in rTMS treatment for PSCI. Some protocols target the left dorsolateral prefrontal cortex (DLPFC), while others focus on the primary motor cortex (M1) [13, 14]. Variations also exist in frequency, intensity, pulse number, and duration. This lack of consensus underscores that the neural mechanisms and optimal stimulation parameters require further investigation. In this review, we summarize the neurobiological mechanisms underlying rTMS in promoting cognition recovery and review the relevant clinical applications of rTMS in PSCI patients.

## 2 rTMS Therapeutic Mechanism in PSCI

### 2.1 Pathogenesis and Progression Factors of PSCI

PSCI is a heterogeneous syndrome characterized by deficits in executive function, memory, attention, language, and visuospatial processing [2]. Compared to stroke patients without cognitive impairment, those with PSCI exhibit significantly higher mortality and disability rates. The pathogenesis of PSCI remains complex and not fully elucidated, involving multiple pathophysiological mechanisms such as hippocampal lesions, white matter hyperintensities, and cerebral microbleeds [3, 15]. Current research identifies PSCI pathogenesis as multifactorial, with significant risk factors including advanced age, cortical infarcts, mesial temporal lobe atrophy, initial stroke severity, a history of recurrent stroke, strategic lesion location, and global cerebral atrophy [16].

Traditionally, AD has been regarded as a neurodegenerative disorder primarily associated with structures like the hippocampus. However, recent research reveals that the hippocampus is also critically involved in the pathogenesis of PSCI, even in cases involving mixed pathology (e.g., stroke superimposed on AD) [17]. For instance, Blum et al. conducted a prospective high‐resolution MRI study of 658 non‐demented elderly individuals and found that the presence of brain infarcts was associated with reduced hippocampal volume. They also observed that smaller hippocampal volume was specifically associated with poorer memory performance. Furthermore, brain infarcts were associated with poorer memory and cognitive performance across other domains [18]. This evidence underscores the hippocampus's role beyond AD, highlighting its significance in vascular cognitive impairment such as PSCI.

### 2.2 Electromagnetic Basis of rTMS Mechanisms

In 1831, Faraday discovered the electromagnetic effect, a major breakthrough in physics that laid the theoretical foundation for TMS. About 150 years later, Barker successfully developed the first functional device capable of modulating cortical neural activity through the application of pulsed magnetic fields [19]. During rTMS treatment, electric current is passed through the coil to generate a magnetic field, which in turn induces an electrical current in the underlying brain tissue. This induced current affects the cerebral cortex, leading to transient enhancement or suppression of cortical neuro‐excitability to modulate intracortical neuronal circuit activity [20]. TMS is categorized into single‐pulse TMS, paired‐pulse TMS, and rTMS based on stimulus pulse patterns. The most predominantly employed modality in clinical practice is rTMS, which involves the delivery of a repetitive pulse train at consistent intensity to a specific cortical region over a defined period. This protocol induces a sequence of neuromodulatory effects, culminating in lasting alterations and regulation of cortical neuronal activity [21]. To date, its clinical application has become increasingly widespread, attributable to the following reasons: (a) It is painless, non‐invasive, and demonstrates a high safety profile with rare side effects; (b) It allows for precise modulation of specific brain regions, maximizing therapeutic accuracy and efficacy; (c) It promotes neural remodeling and reversible modulation of cortical excitability networks, while also enabling simultaneous investigation of brain structure and function [22].

### 2.3 Therapeutic Mechanisms of rTMS in PSCI

rTMS primarily achieves therapeutic effects by modulating cortical excitability and thereby regulating neural circuits. Brain plasticity refers to the brain's ability to reorganize itself by modifying synaptic strength, dendritic remodeling, and neuronal circuit excitability in response to neuromodulation like rTMS [8]. The therapeutic effects of rTMS are determined by a combination of factors including stimulation frequency, duration, number of pulses, site, and train parameters. Generally, high‐frequency stimulation (typically > 1 Hz) is thought to produce excitatory effects, while low‐frequency stimulation (typically ≤ 1 Hz) is thought to produce inhibitory effects [6]. rTMS modulates neurotransmitter systems and regulates proteins involved in synaptic structure and function. It primarily targets Gamma‐aminobutyric acid (GABA) and glutamate, which critically mediate inhibitory and excitatory synaptic transmission, respectively [23]. rTMS also modulates the expression of brain‐derived neurotrophic factor (BDNF), a key regulator of synaptic plasticity. BDNF binds to its receptor, TrkB (Tropomyosin receptor kinase B), activating intracellular signaling cascades that promote neuronal and synaptic growth and differentiation. Additionally, rTMS alters the function of associated receptors, such as NMDA (N‐methyl‐D‐aspartate) and AMPA (α‐amino‐3‐hydroxy‐5‐methyl‐4‐isoxazolepropionic acid) receptors, and modulates the expression of synaptic proteins essential for maintaining synaptic connections, including presynaptic and postsynaptic density protein 95 (PSD‐95) [24, 25].

Moreover, recent studies have found that rTMS can modulate the microenvironment of local neurons in the brain. Cao et al. discovered that 25 Hz rTMS improves cognitive function in mice with vascular cognitive impairment (VCI) by reducing microglial activation and the release of inflammatory factors such as IL‐6, IL‐1β, and TNF‐α [26]. Similarly, Baek and colleagues discovered that high‐frequency rTMS activates the Ca^2+^‐calmodulin‐dependent protein kinase II (CaMKII)‐cAMP‐response element binding protein signaling pathway to reduce oxygen free radical damage and prevent cell apoptosis [27].

## 3 rTMS Therapeutic Effects in PSCI

As a novel neuromodulation approach in neurorehabilitation, transcranial magnetic stimulation (TMS) has been extensively applied in post‐stroke rehabilitation for many years. Its applications include treating various post‐stroke complications such as motor dysfunction, PSCI, post‐stroke depression, and aphasia, with studies demonstrating improved treatment outcomes [28]. To our knowledge, the first documented application of rTMS for improving cognitive function in stroke patients was reported by Jorge and colleagues in 2004 [29]. Although this study did not yield statistically significant cognitive improvement—a result potentially limited by stimulation parameter selection and patient selection criteria—it nevertheless represented pioneering work. Over the past two decades, numerous researchers have employed rTMS to treat PSCI, yielding a body of evidence demonstrating clinically significant benefits. However, current research is predominantly limited to the short‐term effects of rTMS, with a scarce number of RCTs incorporating long‐term follow‐up [13, 30, 31, 32]. Consequently, there is a lack of rigorous evidence to substantiate its potential long‐term efficacy for PSCI. Table 1 summarizes key clinical studies investigating rTMS for cognitive improvement in stroke patients. Subsequently, we will review the therapeutic effects of rTMS across four key cognitive domains: memory function, executive function, attention function, and activities of daily living. Concurrently, we will discuss the most established stimulation targets and parameters employed in these interventions.

**TABLE 1:** Clinical application of repetitive transcranial magnetic stimulation in post‐stroke cognitive impairment.

Study (year)	Participants (*n*)	Study design	Stimulation site	TMS protocol (Frequency, Intensity, Pulse number and Duration)	Outcome measures	Follow‐up time points	Main conclusion
Ricardo E Jorge et al. (2004) [29]	Total 20 rTMS 10 Sham 10	Randomized, parallel, double‐blind trial	left prefrontal cortex	10 Hz, 110% MT, 1000 pulses, 5 days per week for 2 weeks	MMSE; RAVLT; BNT; COWAT	Immediately post‐treatment	No significant improvement
Felipe Fregni (2006) [13]	Total 15 rTMS 10 Sham 5	Longitudinal, randomized, parallel‐design, sham controlled, phase II trial	M1	1 Hz, 100% MT, 1200 pulses, 5 days	MMSE; Stroop test	2 weeks post‐treatment	No significant improvement
Bo Ryun Kim (2010) [33]	Total 18 Low‐frequency rTMS 6 High‐frequency rTMS 6 Sham 6	Double‐Blind, Sham‐Controlled trial	Left DLPFC	1 Hz, 80% MT, 900 pulses 10 Hz, 80% MT, 450 pulses, 5 days per week for 2 weeks	Seoul Computerized Neuropsychological Test; DST	Immediately post‐treatment	No significant improvement
Yuanwen Liu (2020) [34]	Total 58 rTMS 29 Sham 29	Prospective, Parallel, Double‐blinded trial, Randomized Controlled	Left DLPFC	10 Hz, 90% MT, 700 pulses, 5 days per week for 4 weeks	FIM; TMT‐A; DST; DS; MMSE	Immediately post‐treatment	Improved performance in activities of daily living and attentional functioning
Yamei Li (2020) [35]	Total 30 rTMS 15 Sham 15	Prospective, Single‐center, Randomized, Double‐blind, Sham‐controlled trial	Left DLPFC	5 Hz, 100% MT, 2000 pulses, 5 days per week for 3 weeks	MMSE; MoCA	Immediately post‐treatment	Improved cognitive function
Mingyu Yin (2020) [36]	Total 34 rTMS 16 Sham 18	Randomized, Double‐blind, Sham‐controlled trial	Left DLPFC	10 Hz, 80% MT, 2000 pulses, 5 days per week for 4 weeks	MoCA; VST; RBMT; MBI; ADL	Immediately post‐treatment	Improved cognitive function and quality of life
Po‐Yi Tsai (2020) [37]	Total 41 rTMS 11 iTBS 15 Sham 15	Randomized, Controlled, Double‐blind trial	Left prefrontal cortex	5 Hz, 80% MT, 600 pulses; 5 Hz, 3 pulses of 50 Hz bursts, 600 pulses, 5 days per week for 2 weeks	RBANS	1 day post‐treatment	Improved global cognition, attention and memory function
Hong Li (2021) [38]	Total 65 rTMS 33 Sham 32	Randomized, Controlled trial	Contralateral DLPFC	1 Hz, 90% MT, 1000 pulses, 5 days per week for 4 weeks	MMSE; MoCA; MBI	Immediately post‐treatment	Improved visuospatial function, memory, and attention
Fangzhou Yu (2021) [39]	Total 115 rTMS 60 Sham 55	Randomized, Controlled trial	Left DLPFC	10 Hz, 90% MT, 800 pulses, 3 days per week for 8 weeks	MMSE; ADL	Immediately post‐treatment	Improved psychological emotions and cognitive functions
Jongwook Kim (2022) [40]	Total 133 rTMS 49 Sham 84	Randomized, Controlled trial	Ipsilesional DLPFC	20 Hz, 80% MT, 1000 pulses, > 5 times	MMSE; WAIS‐IV; FIM	Immediately post‐treatment	Improved cognitive function
Zhen Zhu (2022) [41]	Total 100 rTMS 50 Sham 50	Randomized, Controlled trial	Left DLPFC	5 Hz, 60% MT, 800 pulses, 5 days per week for 8 weeks	MMSE; WCST	Immediately post‐treatment	Improved neuropsychological function
Bi Yingli (2022) [42]	Total 36 rTMS 18 Sham 18	Randomized, Controlled trial	Unaffected DLPFC	1 Hz, 80% MT, 600 pulses, 5 days per week for 8 weeks	LOTCA; P300	Immediately post‐treatment	Improved cognitive function
Byoung‐woo Cha (2022) [30]	Total 10 rTMS 10	Prospectively designed without control, retrospective trial	Ipsilesional DLPFC	20 Hz, 100% MT, 2000 pulses, 5 days per week for 2 weeks	MMSE; MoCA; WAIS‐IV; AVLT; CFT; MQ; CDR‐SB	Immediately & 12 weeks post‐treatment	Improved cognitive function
Minmin Chu (2022) [12]	Total 60 iTBS 21 tDCS 19 Sham 20	Prospective, Randomized, Single‐blind, and Controlled trial	Left DLPFC	5 Hz, 3 pulses of 50 Hz bursts, 600 pulses; 2.0 mA, 20 min, 5 days per week for 6 weeks	LOTCA; ADL MBI	Immediately post‐treatment	Improved cognitive function and quality of life
Haoran Duan (2023) [31]	Total 71 rTMS 23 Sham A 24 Sham B 24	Randomized, Double‐blind, Sham‐controlled trial	Left DLPFC	10 Hz, 80% MT, 1400 pulses, 5 days per week for 4 weeks	MMSE	Immediately and 8 weeks post‐treatment	Improved cognitive function
An‐Ming Hu (2023) [43]	Total 34 rTMS 12 rTMS‐tDCS 10 Sham 12	Randomized, Controlled trial	DLPFC	5 Hz, 90% MT, 1200 pulses; 1.2 mA, 20 min, 5 days per week for 4 weeks	MoCA; RBMT; MMN	Immediately post‐treatment	Improved cognitive function and dysmnesia
H‐X Zhang (2023) [44]	Total 119 rTMS 61 Sham 58	Retrospective study	Left DLPFC	10 Hz, 80% MT, 1080 pulses, 5 days per week for 4 weeks	MoCA; MBI; P300	Immediately post‐treatment	Restored cognitive function
Kuide Li (2024) [14]	Total 30 rTMS 15 Sham 15	Prospective, Single‐center, Randomized, Double‐blind, Controlled trial	Left DLPFC	10 Hz, 100% MT, 1170 pulses, 5 days per week for 4 weeks	MMSE; P300	Immediately post‐treatment	Improved cognitive function
Hong Li (2024) [32]	Total 31 rTMS 16 Sham 15	Randomized, Controlled trial	DLPFC	1 Hz, 90% MT, 1040 pulses, 5 days per week for 4 weeks	MoCA; VFT; MBI	Immediately and 4 weeks post‐treatment	Improved cognitive function
Jiangping Ma (2024) [45]	Total 73 rTMS 37 Sham 36	Randomized, Double‐blind Controlled trial	M1 and left DLPFC	M1: 10 Hz, 90% MT, 1000 pulses; left DLPFC: 10 Hz, 80% MT, 2000 pulses, 3 days per week for 2 weeks	MoCA; MMSE; ADL; TUG; DTW; FAC, POMA	Immediately post‐treatment	Improved cognitive function
Wei Li (2025) [46]	Total 64 rTMS 33 Sham 31	Randomized, Blinded, Parallel, Sham‐controlled trial	DLPFC	10 Hz, 80% MT, 2000 pulses, 7 days per week for 4 weeks	MoCA; Response time in the n‐back task	Immediately post‐treatment	Improved cognitive function
Xinlin Jiang (2025) [11]	Total 40 L‐rTMS 40 R‐rTMS 40	Randomized, Controlled trial	Left/Right DLPFC	20 Hz, 100% MT, 1000 pulses, 20 consecutive days	MoCA; LOTCA; ADL; P300	Immediately post‐treatment	Left DLPFC more effective improved global cognition
Mengwei Hao (2025) [47]	Total 120 rTMS 120	Randomized, Controlled trial	Left DLPFC	10 Hz, 1080 pulses, 14 consecutive days	MoCA	Immediately post‐treatment	Improved cognitive and neurological function recovery
Xiao Xun (2025) [10]	Total 192 rTMS 100 Sham 92	Randomized, Controlled trial	Prefrontal lobe	10 Hz, 100% MT, 1440 pulses, 5 days per week for 2 weeks	MMSE; MoCA; P300	Immediately post‐treatment	Improved cognitive function

Abbreviations: ADL, Activities of Daily Living; AVLT, Auditory verbal learning test; BNT, Boston Naming Test; CDR‐SB, Clinical Dementia Rating‐Sum of Boxes; CFT, complex figure test; COWAT, Controlled Oral Word Association Test; DLPFC, Dorsolateral Prefrontal Cortex; DS, Digital Span Test; DST, Digit Symbol Test; DTW, Dual‐Task Walking; FAC, Functional Ambulation Category; FIM, Functional Independence Measure; LOTCA, Loewenstein Occupational Therapy Cognitive Assessment; M1, Primary motor cortex; MBI, Modified Barthel Index; MMN, Mismatch Negativity; MMSE, Mini Mental State Examination; MoCA, Montreal Cognitive Assessment; MQ, Memory quotient; POMA, Performance Oriented Mobility Assessment; RAVLT, Rey Auditory Verbal Learning Test; RBANS, Repeatable Battery for the Assessment of Neuropsychological Status; RBMT, Rivermead Behavior Memory Test; TMT‐A, Trail Making Test‐A; TUG, Timed up and Go test; VFT, Verbal Fluency Test; VST, Victoria Stroop Test; WAIS‐IV, Wechsler Adult Intelligence Scale‐IV; WCST, Wisconsin Card Sorting Test.

### 3.1 Stimulation Parameters

The dorsolateral prefrontal cortex (DLPFC) serves as the primary target for rTMS interventions. The summary of Table 1 reveals that the DLPFC was selected as the stimulation target in over 90% of the reviewed rTMS studies for PSCI, with only two exceptions: one study targeted the M1 alone, and another applied concurrent stimulation to both M1 and DLPFC [13, 45]. Within this majority, left‐sided DLPFC stimulation predominates in both research and clinical practice. Functionally, the DLPFC plays a vital role in cognitive control, with particular relevance to post‐stroke recovery of higher‐order functions including working memory, attentional modulation, and executive functioning [40]. The DLPFC constitutes a pivotal hub within the central executive network (CEN), which demonstrates intricate functional associations with multiple cognitive domains—particularly through its robust connectivity with the default mode network (DMN). rTMS targeting the DLPFC modulates activity across both the DMN and CEN, thereby potentially underpinning cognitive improvements [48]. Furthermore, the left DLPFC may represent a more effective target for cognitive enhancement. Jiang et al. demonstrated that rTMS over the left DLPFC improved linguistic and orienting abilities, whereas right DLPFC stimulation enhanced visuospatial functions [11]. The primary motor cortex (M1), primarily known for its role in volitional limb movement control, may also modulate prefrontal and DMN connectivity when stimulated with rTMS. This indirect neuromodulation potentially facilitates cognitive improvement, though its functional efficacy remains comparatively limited relative to direct DLPFC stimulation [13, 45]. The DLPFC is the most common target in PSCI research. This focus is driven by its extensive connectivity with multiple cognitive regions, which underpins the superior clinical efficacy achieved by stimulating this area.

**FIGURE 1:**
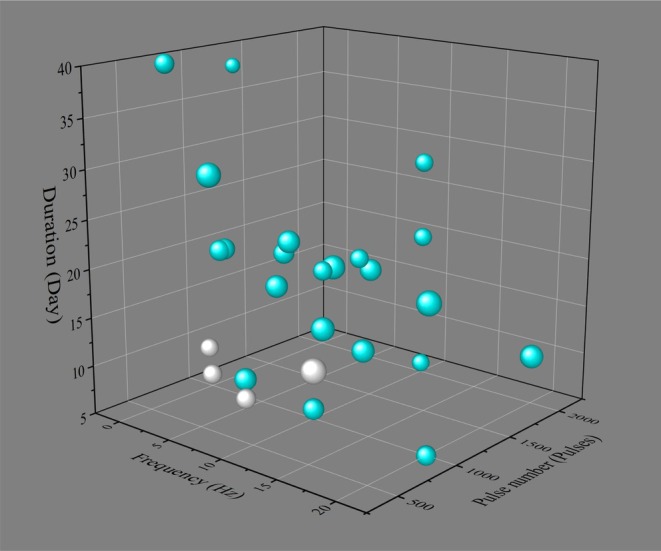
3D Bubble map of rTMS parameters and clinical outcomes in PSCI from randomized controlled trials. Bubble position denotes the combination of frequency, pulse number, and duration. The bubble size scales with stimulation intensity. Light blue bubbles indicate studies reporting significant treatment efficacy, while gray bubbles represent studies with non‐significant outcomes.

Regarding TMS modalities, they are categorized by stimulation pattern into single‐pulse TMS (sTMS), paired‐pulse TMS (pTMS), and rTMS, with rTMS being predominantly employed in clinical practice. Treatment efficacy is determined by a combination of key parameters: frequency, intensity, total pulse number, and treatment duration. However, standardized stimulation parameters have not yet been established for disease‐specific applications (For comprehensive details, refer to Table 1) [49]. Regarding stimulation frequency, frequencies > 1 Hz are conventionally considered to exert excitatory cortical effects, while lower frequencies produce inhibition. In clinical practice, excitatory rTMS protocols with a frequency of ≥ 5 Hz are the most widely adopted. For PSCI treatment, excitatory rTMS protocols are most commonly employed, utilizing frequencies ranging from 5 to 20 Hz. Stimulation intensity is routinely calibrated to 80%–110% of the resting motor threshold (RMT)—defined as the minimal intensity required to elicit motor evoked potentials exceeding 50 μV in the target muscle on ≥ 5 of 10 trials when applied over the motor cortex with the target muscle at rest [3]. Studies exhibit substantial variability in pulse number and treatment duration. It is crucial to recognize that higher pulse numbers do not invariably yield superior therapeutic outcomes. Current protocols typically administer more than 1000 pulses per session, with standard clinical protocols often lasting more than 2 weeks—a common regimen being 20 sessions over 4 weeks. Nevertheless, this rTMS regimen is empirical and must be acknowledged as such. Figure 1 summarizes the parameters of rTMS used for treating PSCI in current studies. Substantial heterogeneity in stimulation parameters is apparent across studies, yet discernible patterns emerge. The visualization suggests that a sufficient total pulse number and treatment duration may be necessary for a therapeutic effect, whereas lower values might be inadequate. The available data indicate that stimulation frequency and intensity may not be the primary determinants of treatment efficacy. We emphasize that this is a preliminary observation from the graphical representation and lacks formal statistical confirmation. The optimal stimulation intensity, number of pulses, and treatment duration have not been defined by dedicated RCTs or meta‐analyses, highlighting a clear need for future studies.

### 3.2 Memory Function

Memory system impairment constitutes a prominent feature of PSCI, with a considerable proportion of patients progressing to dementia, resulting in a significant reduction in quality of life [50]. The efficacy of rTMS in enhancing memory functions has been substantiated by multiple studies, demonstrating improvements in working memory, delayed recall, among other domains. Stimulation targeting the DLPFC elicits therapeutic outcomes, primarily mediated by its structural and functional connectivity with the DMN and frontoparietal network. Facilitation of these neural circuits enhances mnemonic processing through network‐level neuromodulation [51]. However, a meta‐analysis of 10 randomized controlled trials (RCT) indicates that rTMS demonstrates no superior efficacy over controls in enhancing memory function among stroke patients. The observed memory improvements may be attributable to gains in executive function—a hypothesis warranting rigorous investigation given the limited sample size and significant heterogeneity in age and stimulation parameters across included studies [52].

Notably, preclinical evidence substantiates rTMS efficacy, with vascular dementia rat models demonstrating significantly enhanced spatial learning and memory retention in Morris water maze paradigms. Mechanistically, these improvements correlate with upregulated mRNA and protein expression of BDNF, TrkB, and synaptophysin in hippocampal CA1 subfields, suggesting neuroplasticity‐mediated cognitive enhancement [53]. The first documented study of rTMS‐mediated cognitive enhancement was conducted by Jorge et al. in post‐stroke depression patients. Their findings revealed no statistically significant cognitive improvements following intervention [29]. Subsequently, in a cohort of clinically confirmed PSCI patients, Jiang et al. administered 20 Hz rTMS targeting the DLPFC at 100% RMT intensity (1000 pulses/session) across 20 consecutive treatment days. Significant improvements in delayed recall (*p* < 0.05) were documented following active stimulation, as quantified by the Montreal Cognitive Assessment (MoCA) [11]. In a 2020 resting‐state fMRI investigation of rTMS mechanisms in PSCI, Yin et al. recruited 34 patients receiving 10 Hz stimulation at 80% motor threshold (2000 pulses/session; 5 sessions/week over 4 weeks). Longitudinal fMRI data from 14 participants revealed significantly enhanced delayed recall post‐treatment. Crucially, MoCA score changes correlated with amplitude of low‐frequency fluctuation (ALFF) alterations and modified functional connectivity patterns [36]. Collectively, rTMS represents a promising intervention for memory enhancement in PSCI, though its therapeutic potential necessitates definitive validation through large‐scale randomized trials and investigations of synergistic effects with adjunctive pharmacotherapy.

### 3.3 Executive Function

Executive functioning constitutes a paramount and complex component of neurocognitive processing, serving as the foundational substrate for integrated cognition [54]. Executive dysfunction represents a prevalent clinical manifestation of PSCI, characterized by diminished planning/organizational capacities, impaired task execution, and attentional deficits, which substantially compromise patients' quality of life [4]. Crucially, it exhibits an intimate connection with activities of daily living and quality of life metrics, rendering its enhancement an important therapeutic target in PSCI management. Convergent cross‐species evidence identifies robust structural and functional connectivity between the DLPFC and dorsal thalamus as critically implicated in executive functioning [55]. These findings provide mechanistic validation for targeted DLPFC neuromodulation to enhance executive control capacities. Substantial evidence exists documenting rTMS‐mediated enhancement of executive functioning across multiple controlled investigations. Ameis et al. administered a 20‐session, 4‐week course of 20‐Hz rTMS targeting the DLPFC (90% RMT) in autism spectrum disorder patients. Significant improvements in spatial working memory were observed among those with lower baseline functioning. Interestingly, female participants exhibited pronounced gains in this domain. Despite non‐significant global outcomes, these subgroup findings suggest rTMS‐mediated cognitive enhancement potential, necessitating individualized treatment protocols [56]. Although this study was not conducted in a PSCI population, it provides mechanistic support for the ability of DLPFC‐rTMS to modulate executive networks.

Substantial evidence supports rTMS efficacy specifically in PSCI. A meta‐analysis of 20 randomized controlled trials substantiates rTMS efficacy in enhancing executive function among vascular cognitive impairment (VCI) patients. Subgroup analyses identified superior outcomes associated with higher stimulation frequencies, lower intensities, adjunctive comprehensive therapies, and extended treatment durations [57]. In a 2005 pilot randomized crossover trial, Rektorova et al. employed an intermittent theta‐burst‐like protocol: delivered 15 × 10‐pulse trains across three blocks (10‐min intervals) at 100% MT intensity. Significant improvements in the Digit Span Test (DST) and Wechsler Adult Intelligence Scale (IQ) were demonstrated in non‐demented patients with cerebrovascular disease and mild executive dysfunction, indicating early executive function enhancement [58]. In post‐stroke VCI patients receiving rTMS intervention, Cha et al. observed post‐treatment elevation in IQ scores, auditory verbal learning test (AVLT) performance, and complex figure copy test (CFT) accuracy. Concomitant resting‐state fMRI demonstrated activation increases within medial prefrontal cortices, hippocampal formations, and angular gyri. Critically, these improvements persisted at a 3‐month follow‐up, suggesting rTMS may confer durable therapeutic benefits [30]. Liu et al. conducted an exploratory fNIRS investigation in post‐stroke cognitive impairment (PSCI) patients [59]. Participants received a single 10‐Hz rTMS session (90% MT, 900 pulses) with pre/post‐intervention fNIRS during Stroop task performance. Results demonstrated significantly elevated HbO_2_ concentrations in the DLPFC, right premotor cortex (PMC), and right somatosensory motor cortex (SM1) during Stroop tasks versus resting state. Post‐TMS, patients exhibited significantly increased Stroop completion times and error rates alongside enhanced functional activation and strengthened connectivity in these regions. Therefore, while rTMS demonstrates clinically meaningful efficacy in enhancing executive function among PSCI patients, adequately powered, methodologically rigorous RCTs remain imperative to establish individualized treatment protocols.

### 3.4 Attention Function

Attentional capacity serves as a foundational cognitive domain, with deficits representing core symptoms across multiple neuropsychiatric disorders. Attentional deficits constitute a significant contributor to diminished quality of life in PSCI patients, characterized by impaired sustained attention, set‐shifting difficulties, reduced attentional maintenance, and compromised multitasking capacity [60]. The prefrontal and frontoparietal networks are integral to attentional processing and higher‐order cognition. This network architecture engages multiple neurotransmitter systems and structural connectivity patterns, processing information through hierarchical organization across local and large‐scale neuronal ensembles [61]. The DMN plays a core regulatory role in attentional maintenance, exhibiting increased activity during rest and suppressed engagement during focused attention [62]. This neurodynamic principle enables rTMS‐mediated modulation of DMN activity to enhance attentional capacity, with empirical evidence confirming clinically measurable improvements post‐intervention. In Attention Deficit Hyperactivity/Disorder (ADHD) patients, rTMS significantly ameliorates attentional deficits by modulating endogenous dopamine release into the caudate nucleus. A meta‐analysis of 6 RCTs (116 rTMS treated vs. 100 controls) demonstrated significant attention improvement post‐rTMS across stimulation frequencies after adjusting for sex, age, and disease chronicity. Therapeutic efficacy was modulated by stimulus intensity, session duration, and treatment course length [63].

In a cohort of 58 PSCI patients with attentional deficits, Liu et al. augmented cognitive training with adjunctive rTMS (10 Hz, 90% MT, 700 pulses/session, 5 sessions/week × 4 weeks) in the experimental group [34]. Results demonstrated significantly improved performance on Trail Making Test‐A (TMT‐A) and DST relative to cognitive training alone. The intervention group exhibited superior gains across multiple attentional domains including focused attention, sustained vigilance, set‐shifting efficiency, and auditory selective attention, collectively indicating rTMS‐mediated multidimensional attentional enhancement. Furthermore, another study by Liu et al. conducted a randomized controlled trial investigating rTMS efficacy for attentional deficits in PSCI, which also yielded significant improvements [34]. Post‐intervention (1 Hz, 100% MT, 2400 pulses, 5 days per week for 3 weeks), patients exhibited reduced TMT‐A time taken and enhanced accuracy, suggesting substantial enhancement in attentional capacity [64]. Notably, even low‐frequency rTMS stimulation was able to elicit discernible short‐term enhancement of attentional capacity in PSCI patients. Thus, while pharmacological interventions remain the primary treatment for attentional deficits in PSCI, rTMS may represent a compelling non‐pharmacological alternative for targeted attention remediation.

### 3.5 Activities of Daily Living

Activities of Daily Living (ADL) provide an objective measure of functional independence through two hierarchical domains: Basic ADL encompassing self‐care tasks (feeding, dressing, toileting), and Instrumental ADL assessing community interaction skills (shopping, financial management, transportation). Collectively, they serve as primary indicators of functional impairment severity. The ultimate therapeutic objective of rTMS in PSCI is to enhance patients' ADL—the capacity that enables independent home and community functioning and significantly modulates quality of life (QoL). This imperative remains urgent given established evidence that stroke profoundly compromises ADL and QoL, with even minor infarcts exerting detrimental effects [65]. Multiple RCTs have investigated rTMS‐mediated enhancement of ADL in PSCI patients. A meta‐analysis of 2855 PSCI patients demonstrated significant improvements in Barthel Index (BI), Modified Barthel Index (MBI), and Functional Independence Measure (FIM) scores following rTMS intervention, with over 85% of included studies utilizing dorsolateral prefrontal cortex stimulation [66].

Kim et al. conducted a medium‐to‐large RCT enrolling 49 rTMS‐treated and 84 control PSCI patients, assessing ADL via the Functional Independence Measure (FIM). Results demonstrated significant FIM score improvements at 1 month post‐intervention across both left and right hemispheric lesion subgroups following at least five treatment sessions [40]. Liu et al. assessed functional independence using the Functional Independence Measure (FIM), demonstrating significant post‐intervention improvements in total scores as well as motor and cognitive subscale scores following rTMS treatment [34]. Adjunctive pharmacotherapy may potentiate rTMS efficacy. Yu et al. randomized PSCI patients to fluoxetine monotherapy versus combined fluoxetine and rTMS (10 Hz, 90% MT, 800 pulses/session, three sessions/week for 8 weeks). The combination group demonstrated significantly greater BI improvement versus pharmacotherapy alone, though moderate gains occurred with fluoxetine monotherapy [39]. Current evidence indicates that rTMS enhances ADL in patients, yielding measurable improvements in both motor and cognitive functional domains. As a non‐pharmacological neuromodulation approach, rTMS represents a promising therapeutic tool for PSCI, with potential for augmented efficacy when integrated with pharmacotherapy or complementary medicine modalities such as acupuncture and traditional Chinese medicine.

### 3.6 Combined rTMS and Rehabilitation

Currently, rTMS is increasingly combined with other interventions, such as medication, acupuncture, physical therapy, and cognitive training, to more effectively promote cognitive recovery in patients [3, 34, 47, 67, 68]. Donepezil and memantine are the most commonly used drugs for PSCI. Although they provide some benefit, growing evidence suggests that combining pharmacological therapy with rTMS may yield superior outcomes. However, no studies have specifically investigated combining rTMS with donepezil or memantine for PSCI. Ginkgo diterpene lactone meglumine injection—another agent proven to aid cognitive recovery post‐stroke—has been evaluated in combination with rTMS, demonstrating significant cognitive improvement in PSCI patients [47]. Furthermore, traditional Chinese acupuncture has also been recognized as a therapeutic option for PSCI [68]. Xun et al. conducted a study involving 192 PSCI patients, comparing rTMS monotherapy against its combination with Xingnao Kaiqiao acupuncture. After a 4‐week intervention, both groups exhibited significant cognitive improvement, as evidenced by increased MMSE and MoCA scores. It is noteworthy that the combined therapy group demonstrated superior efficacy [10]. Cognitive training represents another approach to improving cognitive function [34]. Commonly adopted cognitive training paradigms include memory training, attention training, orientation training, visual–spatial perception training, reasoning and judgment training, and executive function training. A meta‐analysis of 8 studies indicated that rTMS combined with cognitive training significantly enhances cognition and activities of daily living in PSCI patients; however, the evidence remains limited, with no significant improvement observed in memory specifically [69]. In conclusion, while rTMS has been validated as an effective adjunctive therapy for cognitive impairment in PSCI, evidence supporting its combination with other treatments remains limited. This is particularly true for its combination with first‐line PSCI medications, which lacks dedicated clinical studies. Therefore, future large‐scale, multicenter RCTs are needed to provide high‐level evidence.

## 4 Safety Assessment of rTMS

rTMS is widely regarded as a relatively safe, non‐invasive treatment for PSCI. However, it is not without potential risks. The most common adverse effects include headache, scalp discomfort, and facial twitching [6, 11]. These reactions are typically mild and transient, causing no persistent injury, and usually subside shortly after stimulation ceases. Qiao et al. conducted a meta‐analysis which included 273 patients, 12 of whom experienced the aforementioned complications. Interestingly, four of the included clinical studies reported no adverse reactions [70]. Seizure induction is the most serious potential complication of rTMS therapy and a primary safety concern. The 2018 expert consensus conference of the International Federation of Clinical Neurophysiology concluded that the risk of rTMS‐induced seizures is very low, with approximately just over 40 cases reported to date [49].

Therefore, the following points need to be observed during clinical practice: (1) TMS should be used with caution in patients with metal or other material implants to avoid potential overheating induced by TMS and implant malfunction [21]. (2) Operators must be properly trained and have emergency resuscitation equipment readily available to manage severe complications such as seizures. (3) Particular caution is warranted in patients with epilepsy or certain psychiatric disorders. A careful review of the literature and preliminary safety testing are advised to determine appropriate stimulation parameters prior to treatment. Continuous clinical monitoring is essential during sessions [71]. (4) To minimize occupational exposure, operators should maintain a safe distance from the magnetic coil and wear appropriate hearing protection to mitigate long‐term exposure to magnetic fields and acoustic noise [49].

## 5 Conclusion

rTMS has emerged as a promising, non‐invasive therapeutic modality for PSCI. Accumulating evidence from both preclinical and clinical studies indicates that rTMS can promote cognitive recovery, particularly in the domains of memory, executive function, attention, and ultimately, ADLs. The therapeutic mechanisms are multifaceted, primarily involving the modulation of cortical excitability, the enhancement of neuroplasticity through BDNF‐related pathways, and the regulation of dysfunctional brain networks, notably the CEN and DMN. The DLPFC is the most frequently targeted region, with excitatory high‐frequency protocols being predominantly employed. Although current findings are encouraging, significant heterogeneity in stimulation parameters (e.g., frequency, target, dosage) across studies underscores the urgent need for standardized, optimized treatment protocols. Currently, there is limited evidence regarding the long‐term efficacy of rTMS for PSCI. Future studies should incorporate extended follow‐up periods to evaluate potential delayed effects. Furthermore, while rTMS demonstrates a favorable safety profile, strict adherence to established guidelines is imperative to mitigate risks. Future research must prioritize large‐scale, well‐designed randomized controlled trials with long‐term follow‐up to definitively establish efficacy, optimize individual treatment parameters, and explore synergistic effects with pharmacotherapy or other rehabilitation strategies.

## Author Contributions

Yu L.: Conceptualization, methodology, writing – original draft, writing – review and editing; Yansong L., D.D.: Writing – original draft, writing – review and editing; A.X., J.Y., X.X., Z.Z., J. Wu: Methodology, writing – original draft; J. Wang, M.Q.: Conceptualization, writing – original draft, writing – review and editing, funding acquisition, resources, supervision.

## Conflicts of Interest

The authors declare no conflicts of interest.

## Data Availability

The data that support the findings of this study are available from the corresponding author upon reasonable request.
